# 

**Published:** 1852-11

**Authors:** 


*Receipts of Medical Journal for October,* 1852.

| | |
|---|---|
| Dr. J. K. Winlock, Evansville, Ind., - - - - - | $5 00 |
| W. R. Chew, Midway, Ky. - - - - - - | 5 00 |
| R. Scruggs, Shreveport, La., - - - - - | 5 00 |
| V. T. West, Union, Ind., - - - - - | 10 00 |

